# Association of mode of entry to a nursing programme and student success: A two-year retrospective multi-cohort study

**DOI:** 10.4102/hsag.v29i0.2560

**Published:** 2024-04-23

**Authors:** Fransisco C. Ntjamba, Daniel O. Ashipala, Yahaya Jafaru

**Affiliations:** 1 Department of General Nursing Sciences, Faculty of Health Sciences and Veterinary Medicine, University of Namibia, Rundu, Namibia; 2 Department of Nursing Science, College of Health Sciences, Federal University Birnin-Kebbi, Kebbi State, Nigeria

**Keywords:** mature-age entry, nursing programme, student success, mode of entry, undergraduate

## Abstract

**Background:**

The issue of the mode of entry to nursing programmes and its effect on student success is a key concern among researchers globally. Identifying the mode of entry, which has the potential to improve academic success, decrease the failure rate and lead to the successful completion of a degree, is crucial to increase the nursing workforce.

**Aim:**

The objectives of this study were to assess the association of mode of entry to a nursing programme on student success among undergraduate students.

**Setting:**

The study was conducted at a public nursing education institution (NEIs) in the northeast of Namibia.

**Method:**

A retrospective multi-cohort study was conducted to assess the association of mode of entry on student success. Academic outcomes were analysed, observing two cohorts of nursing students.

**Results:**

Results show that 76.2% (f = 16) of mature-age entry students and 53.7% (f = 29) of direct entry students completed their programme on time. However, 42.1% (f = 8) of access students were expected to return. Female students 56.8% (f = 25) and male students 56.0% (f = 28) completed on time. There was no significant relationship between the mode of entry and completion status with *p* > 0.05, respectively.

**Conclusion:**

A higher percentage of mature-age entry students was found to complete their studies on time than direct entry and English access entry students.

**Contribution:**

These findings could be used in the revision of student recruitment strategies to select nursing students who are more likely to achieve the best academic outcomes.

## Introduction

The debate on the selection of suitable candidates to deliver nursing, pass professional body examinations and practice nursing competently has become an issue of concern (Mthimunye & Daniels 2019). This is because suitable candidates produce suitable students and having suitable nursing students provides the nursing profession with improved future manpower and enhanced patient safety (Gale et al. 2016). Moreover, the selection of the right candidates is related to an increase in student success and a decrease in the attrition rate. Ogbonnaya et al. (2014) emphasised that the selection of the right candidates to read nursing science is a fundamental step in producing a large number of graduates at the end of the programme. The selection is also crucial for developing quality nurses because the development of the profession is dependent on the kinds of individuals enrolled (Sim et al. 2018).

Academic success in nursing education means progression through nursing courses with at least the minimally prescribed passing grades and progression through the curriculum to graduation (Betts, Shirley & Kennedy 2017). The issue of nursing students’ success has received increased attention in recent years, largely owing to growing concerns about the diminishing quality of nursing care, high attrition rates, limited resources and students’ academic failure (Smith, Engelke & Swanson 2016). The nursing students’ success highlights the importance of modes of entry as these can be used to identify and admit candidates with higher potential for success (Patterson, Griffin & Hanson 2018). It also prompted thoughts about the effects of mode of entry on student success, leading to an extensive debate among many academics and aspiring researchers to find out what mode of entry is the best predictor of student success (Richard-Eaglin 2017). Mode of entry in this study refers to a method accepted by the university through which a student secures admission to the University to read Nursing Science. The modes of entry for the University of Namibia (UNAM) include direct entry admission, access course, recognition of prior learning, science foundation, mature-age entry candidates and candidates from the Namibia College of Open Learning (NAMCoL).

Dube and Mlotshwa (2018) state that currently the academic failure of nursing students is an issue of great concern and international interest, owing to its negative effects on the availability of future nurses in different aspects of health care systems, affecting the global economy. There are still many challenges related to student retention and success encountered by nursing schools and the academic failure rate of students in nursing programmes ranges from 9% to 46.3% globally (Lancia et al. 2018). However, Paton (2018) and Rogers (2017) assert that the attrition rate of students admitted to nursing programmes who do not reach graduation, ranges between 25% and 50%. There are also reports of students dropping out of university nursing programmes either voluntarily or involuntarily (Ten Hoeve et al. 2017; Wray et al. 2017).

The purpose of admission strategies for nursing students includes admitting students who have the potential for timely progression (Tartavoulle et al. 2018). The admission plans allow nursing education institutions (NEIs) to examine the effectiveness of their various methods for admitting students (Al-Alawi 2020). Thus, there are calls to revisit the selection criteria of nursing institutions to limit the time students remain in the nursing programme and to make way for the production of more qualified nursing professionals (Ndwambi & Roets 2020). Many researches on this effect has concentrated only on cognitive variables even though there is consensus that both cognitive and non-cognitive variables are important for admitting students with high success potential (Al-Alawi 2020).

Considering the above situation and with the need for selection strategies to consider candidates with higher success potential (Ndwambi & Roets 2020), assessing the effects of mode of entry on students’ success may assist NEIs in identifying candidates who are likely to face challenges and may spend extra years of study in nursing programmes (Saliu & Musa 2017). Gale et al. (2016) also assert that, unfortunately, there is a lack of evidence backing most admission criteria. Al-Alawi (2020) maintains that research studies on variables associated with or which predict success among students in nursing programmes are still relevant and vital. The selection of candidates with a high potential for success can only happen if research studies examine all the selection methods critically. This study therefore assessed the association between modes of entry to a nursing programme and student success in the form of a 2-year retrospective multi-cohort study.

Selecting students who can successfully complete their education and gain the required professional qualification is considered a major challenge by higher education institutions around the world (Gale et al. 2016). The global shortage of nurses has created a gap in caring for the sick and injured (AACN 2019; Paton 2018) because the new annual nurse supply is always less than the demand (AACN 2019). The successful completion of all the students admitted to a nursing programme cannot be guaranteed. The high rate of student dropouts in Nursing Science programmes is likely because of ineffective admission policies that fail to identify potentially unsuccessful students (Elkins 2019). One of the major challenges in nursing education is the selection of competent candidates who are most likely to complete the training programme successfully and make an effective long-term contribution to their profession, the general public and the community (Smiley et al. 2018).

In Namibia, interest in studying nursing has increased tremendously in recent years in response to the shortage of nurses, but academic success among nursing students remains a major concern. The Namibia National Council for Higher Education enables universities in the country to permit various entry pathways for interested candidates to enter a specific programme, especially nursing.

The UNAM is the premier institution of higher education in the country, which makes a study on the association of entry to nursing programmes on student success at UNAM pertinent. The preceding several decades have seen a scarcity of nurses in the job market, which has sparked interest in nursing education. As a result of the call, the number of nurses in the nursing field increased tremendously, and there was a rapid response to the nursing shortage. Poor academic performance among nursing students has been a major concern in recent years. One of the identified factors influencing academic performance could be the mode of entry into nursing program. There are basically six modes of entry into nursing program, namely: Mature age entry, science foundation learning, English access course, direct entry, recognition of prior learning and students from NAMCoL. Therefore, studying students’ academic outcomes and their potential determinants could be the first step for universities to face students’ difficulties and consequently facilitate the nursing students’ academic success, promoting their own economic sustainability and contributing to satisfy the community’s health needs (Jeffreys 2015). However, even though much evidence showed that students’ profiles, including gender, age and pre-entry qualification, could be associated with academic outcomes, such results do not allow the adoption of effective strategies to reduce academic failure as they were often conflicting or focused on non-modifiable students’ characteristics (Dante, Petrucci & Lancia 2013). Nevertheless, the entry-test scores and upper-secondary school grades have often been identified as good criteria to select the best candidates (Lancia et al. 2013) even though in the nursing field the contrasting evidence highlights the need to deeply understand the role of these predictors (Dante et al. 2013).

There are various requirements for direct entry admission, access course, recognition of prior learning, science foundation, mature-age entry candidates and those from NAMCoL. Namibia National Council of Higher Education enables its universities to permit various entry pathways for interested candidates to enter a specific programme, especially into the nursing program. Two years after Namibian independence, the University was founded on 31 August 1992. One of the take-up programmes is the Bachelor of Nursing (14BNCL), which made the University a pioneer university that trains nurses up to degree level in the whole country. As a result, qualified nurses have now increased and widely spread at the national level, and this allows the researcher to conduct a study on the issue of mode of entry and performance at the UNAM.

The modes of entry used by the UNAM have different criteria. Firstly, the Mature-Age Entry is an alternative admission route for prospective students not in possession of a Grade 12 Certificate or for those with a Grade 12 Certificate who do not fulfil the minimum admission criteria based on Grade 12. Secondly, Direct Entry are those candidates who have not attended a post-secondary institution or have completed fewer than 24 credit hours at a recognised university or college. Thirdly, the English access entry is a 1-year full-time English Access Course designed for students who have reached the minimum required points for admission to UNAM, except for the required minimum C grade in English. The course is aimed at bridging the students’ English symbol to be at par with the minimum requirements for UNAM. The course is designed for students who have completed Grade 12 with a minimum of 25 points (that is, top four subjects should be 22 points plus English with an E or D-symbol). Fourthly, NAMCOL is a State-Owned educational institution created by an *Act of Parliament (Act 1 of 1997)* to provide learning opportunities for adults and out-of-school youth to improve their grade 12 and qualify for universities. Therefore, NAMCOL offers the programme to Grade 12 learners who did not perform well in the first examination sitting and wish to improve their grades for admission at Institutions of Higher Learning such as UNAM. Fifthly, the Recognition of Prior Learning (RPL) refers to a broad spectrum of processes, all aimed at reaching a judgement of an individual’s personal particulars, current competencies, work and life experience and formal and/or non-formal learning, assessed against transparent criteria for a variety of purposes. Recognition of Prior Learning allows experienced adults to become qualified based on what they already know and what skills or competences they already possess. Finally, the sixth and last entry scheme is the Science Foundation Programme, which is a 1 year face-to-face full-time, bridging course aimed at strengthening skills and understanding of former Grade 12 learners in English, Physics, Biology, Chemistry and Mathematics. The programme refers to the processes through which the knowledge and skills a person acquired previously are measured and assessed for the purpose of formally recognising such achievements, and the prior learning would be assessed against one or more unit standards or components of a part-qualification or qualification.

In the context of this study, complete on time means finishing the given course within the prescribed number of years. Expected to return refers to those students who failed some educational modules and are required to repeat the module the following year. There are different admission requirements based on the mode of entry. However, except for the mature-aged entry that simply requires the student to be 25 years or more, work experience in a field applied for and having passed Grade 10, as well as the RPL, the rest have a common denominator of obtaining at least 25 points in five subjects and at least a D in English regardless of the processes involved.

## Research methods and design

### Study design

A retrospective multi-cohort study was conducted and reported according to the Strengthening, the Reporting of Observational Studies in Epidemiology (STROBE) recommendations (Cevallos et al. 2014). Two cohorts of undergraduate students admitted to the Bachelor of Nursing Science (BNSc) degree in two academic sessions in 2017 and 2018 were included.

### Population of this study

The population of this study comprised nursing students who had successfully registered for a bachelor’s degree in the 2017 and 2018 sessions. The study was conducted at one of the satellite campuses of UNAM located in the Kavango East region (Rundu Campus).

### Research instrument

The data of the study were collected with the aid of using a checklist of students’ information containing demographic data and their academic success in terms of dropouts, failure and graduate rates or completion status as this was used by the researcher to serve as an instrument for the study. The data from the checklist were used to determine the effects of mode of entry on student success with the analysis of chi-square and multinomial regression analysis.

### Data collection

The entire list of students registered for 2017 and 2018 was obtained from the department concerned after obtaining permission from the department management. All students with complete information on their records were selected. Subsequently, 94 students were found to be eligible for selection. The data collected from the student records included the mode of entry and programme completion status. Modes of entry included mature-age entry, direct entry and English access entry. The completion status includes completed on time, expected to return and completed on duration. The students with a ‘complete on time’ status mean that the Nursing course was done within the 4-year period, while the ‘expected to return’ status refers to the students who have failed in their fourth year of study and are yet to continue with their studies. The ‘completed-on duration’ status applies to the students who have not completed within the minimum 4 years of study; instead, they complete within the 6 years, which is the allowable maximum number of years a student is allowed to be enrolled in a Nursing programme. The completion status is used as information pertaining to students’ success.

### Data analysis

Data were analysed using SPSS version 26. Frequency and percentages were used in analysing the characteristics of the respondents, while chi-square was applied in determining the relationship between mode of entry and completion status. Internal consistency reliability was determined using Cronbach’s alpha coefficient (α) for all scales of determining the relationship between mode of entry and completion status. A one-sample *t*-test was applied to test for the significance of agreement or disagreement for each of the two variables. Pearson product-moment correlation coefficient was used to determine the association mode of entry and student success. Throughout, a *p*-value of 0.05 was used to indicate the significance.

### Ethical consideration

Approval for this study was granted by the university of University of Namibia’s School of Nursing and Public Health Research Ethics and Review Committee (SoNPHREC) (reference no 136/2022). Participant consent was not obtained because this study had no participants.

## Results

The study consisted of 94 students chosen randomly from a cohort of 2017 and 2018 academic sessions collected in the subgroups of direct entry, mature-age entry and English access. Table 1 shows the characteristics of the study subjects.

**TABLE 1:** Characteristics of the study subjects (*N* = 94).

Variables	Frequency	%
**Mode of entry**		
Mature-age entry	21	22.3
Direct entry	54	57.4
English access	19	20.2
**Gender**		
Male	44	46.8
Female	50	53.2

The majority (57.4%) of the study subjects were direct entry students and 22.3% were mature-age entry. This is illustrated in Table 1. In terms of gender, 53.2% of the study subjects were female and 46.8% were male.

In order to assess the completion rate as an academic success, there was a need to analyse the mode of entry for the two cohorts under study as shown in Table 2.

**TABLE 2:** Descriptive results of the study: Subjects’ year enrolled and the mode of entry cross-tabulation (*N* = 94).

Crosstabulation	Cohort	Frequency Percent	Mode of entry			Total
			Mature age entry	Direct entry	English access	
Year study begins	Cohort 2017	Count	14	40	10	64
		% within year study begins	21.9	62.5	15.6	100.0
		% of Total	14.9	42.6	10.6	68.1
	Cohort 2018	Count	7	14	9	30
		% within year study begins	23.3	46.7	30.0	100.0
		% of Total	7.4	14.9	9.6	31.9
Total		Count	21	54	19	94
		% within year study begins	22.3	57.4	20.2	100.0
		% of Total	22.3	57.4	20.2	100.0

The results depicted in Table 2 show that the majority 62.5% of students who enrolled in 2017 were through direct entry, while 21.9% were through mature-age entry. The same observation was made for direct entry of those who were admitted in 2018 with a representation of 46.7%. Overall, more than half of the admissions into the nursing programme in 2017 and 2018 were through the direct entry mode 57.4%.

The study subjects’ completion status was based on the time taken to complete the Nursing program. Subjects who completed on time took 4 years to finish, those who completed on duration took 6 years to finish, while those who were expected to return have covered the 4 years; however, they have not completed the studies and are within the 6 years of enrolment. The results on this aspect are presented in Table 3.

**TABLE 3:** Completion status of the study subjects (*N* = 94).

Variables	Frequency	%
Completed on time	53	56.4
Expected to return	28	29.8
Completed on duration	13	13.8

Table 3 illustrates the level of completion achieved by the study participants. A total of 56.4% of the subjects successfully finished the programme within the designated timeframe, while 29.8% were expected to return. Only 13.8% managed to complete the programme within the expected duration.

The association between the mode of entry and the academic success or completion rate was analysed using a chi-square test statistic. The results are shown in Table 4.

**TABLE 4a:** Relationship between mode of entry and completion status (*N* = 94).

Variables	Mature-age entry	Direct entry	English access
	F (%)	F (%)	F (%)
Completed on time	16 (76.2)	29 (53.7)	8 (42.1)
Expected to return	5 (23.8)	15 (27.8)	8 (42.1)
Completed on duration	0 (0.0)	10 (18.5)	3 (15.8)
**Total**	**21**	**54**	**19**

Note: Table 4a shows that 76% of mature-age entry students and 53.7% of direct entry students completed their programme on time. In addition, 23.8% mature-age entry students and 27.8% direct entry students were expected to return, while 42.1% of English access students were expected to return.

**TABLE 4b:** Chi-square tests.

Statistic	Value	*df*	Asymp. sig. (2-sided)
Pearson chi-square	7.325a	4	0.120
Likelihood ratio	9.975	4	0.041
Linear-by-linear association	3.541	1	0.060
Number of valid cases	94.000	-	-

Note: Table 4b shows that there was no significant difference between the mode of entry and completion status, chi-square = 7.325, *df* = 4, and *p* = 0.120 at 0.05 level of significance.

Sig., significance; *df*, degrees of freedom

Table 4 shows that 76% of mature-age entry students and 53.7% of direct entry students completed their programme on time. In addition, 23.8% mature-age entry students and 27.8% direct entry students were expected to return, while 42.1% of English access students were expected to return. The same table shows that there was no significant difference between the mode of entry and completion status, chi-square = 7.325, *df* = 4 and *p* = 0.120 at 0.05 level of significance.

To determine the correlation between the mode of entry and the completion status, the Pearson correlation was used, and the results are shown in Table 5.

**TABLE 5:** Correlations between study subjects’ mode of entry and completion status (*N* = 94).

Variables	Statistic	Mode of entry	Completion status
Mode of entry	Pearson correlation	1.000	0.195
	Sig. (two tailed)	-	0.059
	*N*	94.000	94.000
Completion status	Pearson correlation	0.195	1.000
	Sig. (two tailed)	0.059	-
	*N*	94.000	186.000

Sig., significance

Table 5 informs that the Pearson correlation is 0.195, which is not far from 0. The results indicate that there is no linear relationship between the two variables and there is no consistent pattern or connection between the mode of entry and completion status.

## Discussion

Ascertaining nursing students’ success in terms of completion of the programme is very important for nursing programmes and the profession at large. Connie (2020) highlighted the necessity of evaluating the factors affecting nursing students’ success for the purpose of helping those in need of academic support. It is evident in the findings of this study that different modes of entry to a nursing programme can have an impact on student success and completion rates. The results show that mature-age entry students had a higher percentage of completing their studies on time compared to direct entry and English access entry students. These findings align with previous research conducted by Al-Alawi (2020), who also found that certain factors can influence nursing students’ academic success. However, it is important to note that the study by Connie (2020) used different entry criteria and had variations in sample size and other variables associated with the schools and students, which could explain the differences in findings.

Improving nursing programme success not only benefits the school and the students but also the people that nursing graduates serve (Al-Alawi 2020). The current study found that over half of the respondents had on-time completion of the programme. However, it is worrying to note that over two-fifths of the subjects were unable to complete on time, which could lead to an increased burden for nursing staff resulting from a nursing shortage. Moreover, finding solutions to the growing needs of the nursing workforce rests on nursing programmes (Blitchok 2018); for the purpose of remediating this situation, it is important to be aware of the factors contributing to successful completion and those hindering it. Alexander (2019) suggests that universities should investigate the indicators of nursing quality and outcomes. Thus, this responsibility rests on policymakers governing the nursing programmes, nurse educators and researchers. In addition, Jeffreys (2015) pointed out the necessity of understanding the interactions between student characteristics and student success.

Over half of the mature-age entry and direct entry students completed their programme on time, while those who had English access entry comprised less than half of the students who completed the programme on time. Moreover, mature-age entry makes up the lowest percentage of expected to return compared to direct entry and English access entry students. This is an indication that mature-age entry candidates are more closely associated with graduating on time than direct entry and English access entry candidates. These findings are crucial as educational health programmes, including nursing programmes, are facing a shift in student admission procedures, taking a holistic approach that includes non-academic variables (Wambuguh, Eckfield & Hofwegen 2016). However, limited action has been taken to expedite this shift (Al-alawi 2020). Understanding the mode of entry with a high potential for on-time success could be of great assistance in helping NEIs to select appropriate candidates.

In support of the findings of this study, Al-Alawi (2020) conducted a mixed methods study that explored the factors influencing nursing student academic success. The study found that factors such as age, prior health care experience and motivation were positively associated with academic success. These findings further emphasise the potential benefits of mature-age entry students in terms of their higher completion rates. On the other hand, it is important to consider contrasting viewpoints and studies that may provide a different perspective. Alexander (2019) discusses the evaluation of nursing education programmes and highlights the need for a comprehensive assessment that includes multiple dimensions of success, such as clinical competence and critical thinking skills. This suggests that the mode of entry alone may not be the sole determinant of student success and other factors should be considered when evaluating nursing programmes. The study by Connie (2020) used Health Education Systems, Inc. (HESI) Admission Assessment Exam (HESI-A2) students’ results as criteria for obtaining admission, while the current study used different entry criteria. This, together with the differences in sample size and other compounding variables associated with the schools and students such as teacher–student interactions and students’ economic background, could have been the reasons for the differences.

### Strengths and limitations

This study used a multi-cohort design that allows for comparisons across different groups of students. This design can provide a more comprehensive understanding of the relationship between modes of entry and student success. Additionally, a retrospective study design can provide valuable insights by analysing data from past cohorts of students. The study was limited to a retrospective method, which relies on existing data, which may be incomplete or have missing information. This can introduce bias and limit the conclusions that can be drawn. Another limitation is the potential for confounding variables, which are factors that may influence both the mode of entry and student success. Controlling for these variables can be challenging in a retrospective study.

## Conclusion

In this study, a higher percentage of mature-age entry students was found to complete their studies on time than direct entry and English access entry students. In addition, direct entry and English access entry students formed the highest percentage of those expected to return. Thus, mature-age entry would appear to have an advantage in completing on time over other modes of entry; this category is also less associated with the category ‘expected to return’ than other modes of entry. Male and female students displayed very similar percentages in on-time completion but male students had an advantage in terms of completion on time and expected to return. These findings could be used in the revision of student recruitment strategies in order to select nursing students who are more likely to achieve the best academic outcomes and in providing an intervention that would be helpful to those students unable to complete the study on time.
